# Editorial: Advances and Trends in Polyhydroxyalkanoate (PHA) Biopolymer Production

**DOI:** 10.3389/fbioe.2022.873250

**Published:** 2022-04-19

**Authors:** Justyna Mozejko-Ciesielska, Prasun Kumar, Paulo Costa Lemos, Youwei Cui

**Affiliations:** ^1^ Department of Microbiology and Mycology, Faculty of Biology and Biotechnology, University of Warmia and Mazury in Olsztyn, Olsztyn, Poland; ^2^ DBT-IOC Centre for Advanced Bioenergy Research, R & D Centre, Indian Oil Corporation Ltd., Faridabad, India; ^3^ LAQV-REQUIMTE, Department of Chemistry, Nova School of Science and Technology, Universidade NOVA de Lisboa, Caparica, Portugal; ^4^ Faculty of Environment and Life, Beijing University of Technology, Beijing, China

**Keywords:** high cell-density culture, polyhydroxyalkanoates, pure/mixed cultures, biodegradable polymer, co-polymer

Plastics are key materials in delivering many benefits to society. Possessing a high level of strength, durability and lightness they have become versatile and innovative materials used in numerous products from nearly all industrial sectors. However, due to low degradability their production has led to serious environmental problems. Worldwide production of plastics is still increasing reaching 367 million tons in 2020, so 163 million tons more compared to 2002. It was estimated that in 2020 over 23% of plastic post-consumer wastes were discarded in the landfills (Plastics Europe, 2021). During their decomposition under the actions of biological, physical, and chemical processes, microplastics are formed which nowadays are a major global concern due to their threat to wildlife and humans. These small plastic particles have spread globally to even the most remote habitats becoming a new pollutant and one of the most important scientific issue in environmental science (Zhang et al., 2021). Furthermore, not only problems related to the utilization of plastic waste but also new regulations for its use and management, together with increasing concerns about global warming and climate change are reasons to search for biobased substitutes for petroleum-derived polymers. Moreover, the increased demand for sustainability on the utilization of plastics opens a new opportunity for a myriad of biopolymers that are generated in microbial processes. Over the past decades, great attention has been focused on polyhydroxyalkanoates (PHAs), environmentally friendly polymers with plastic-like properties.

The focus of this Special Issue is to highlight the latest advances in the microbial synthesis of PHAs. It addresses several distinguished aspects of the PHA production chain related to the development and design of novel bioprocesses towards the improvement of PHAs production, description of microbes as PHAs producers of industrial interest, molecular design of polymers with a variety of properties, solutions to decrease overall production costs of microbial PHAs.

This special issue consists of a total of six original articles, one brief research report, two specialized review papers, and one systematic review written by globally recognized experts who have had a significant impact on this research area already for a long time.

In the early 1990s, an exponential burst of scientific publications concerning research on PHAs started. Since then, different areas of exploration of these intriguing materials have been uncovered. However, to understand the field of these studies a systematic literature review is essential. Guzik et al. have performed an unbiased search of up-to-date literature to reveal trending topics in the research of PHAs over the past 3 decades by data mining of 2,227 publications. The conducted analysis allowed to identify eight main areas in PHA research that governed the 31 years of discoveries. The analysis of the articles published from 1988 to 2019 provides a comprehensive review of the trends and speculates where PHA research is heading.

In an attempt to increase PHA production, PHB biosynthesis operon of alkali tolerant *Cupriavidus necator* strain A-04 was expressed in *Escherichia coli* under the control of cold shock promoter (*cspA* promoter) by Boontip et al. The PHA synthase remained insoluble in the tested expression systems until it was co-expressed with TF chaperones. This study revealed that the *cspA* promoter in a cold-inducible vector can enhance PHA synthase expression and solubility without affecting polymer properties. The induction strategy was demonstrated on a 5-L fermenter showing a PHB production of 7.9 g/L with 89.8% PHB content in the cell dry mass. In another study, Napathorn et al. tested the expression system consisting of *araBAD* promoter and thioredoxin fusion proteins and compared it with the cold shock inducible expression system. This work demonstrated that both expression systems were able to yield high cell density and PHB production in fed-batch cultivation using a pH-stat feeding strategy. It was proposed that arabinose-based induction system would be a cheaper alternative to IPTG. Thus, *E. coli* XL1-blue expressing pBAD/Thio-TOPO-*phaCAB*_A–04_ and pColdTF-*phaCAB*_A–04_ possess a high potential for PHB production.

In the context of the potential to expand the molecular design of polymers with a variety of properties Arai et al. shows the capacity to incorporate glycolate (GL) units into the chains of PHAs since this monomer is not present in PHAs of natural occurrence. For that a new chimeric PHA synthase (PHACAR) containing the N-terminal region of PhaCAc derived from *Aeromonas caviae* and the C-terminal region of PhaCRe derived from *Cupriavidus necator* was constructed. This new class I PHA synthase was expressed together with propionyl-CoA transferase (PCT) in *Escherichia coli*. This microorganism was able to produce a random-homoblock PHA, P (GL-ran-3HB)-b-P (3HB) for the first time. The produced polymers were analyzed in terms of their thermal properties, solvent fractionation, and sequence heterogenicity. The simultaneous presence of random and block sequences in this biopolymer can be useful to control the physical properties of each segment and so to design new polymers with an array of different characteristics. The incorporation of the GL units can potentially manipulate the bioabsorption rate of PHAs, as hydrolytically degradable material, useful in biomedical fields.

Substrate cost contributes towards a major portion of incurred cost during PHA production. Several wastes have been explored for their suitability and potential for PHA production. Due to the flourishing biodiesel industry across the globe, the surplus amount of crude glycerol has emerged as a suitable feedstock for the bio-production of industrially important chemicals. In this context, algal biodiesel waste residue (source of crude glycerol) was tested as a substrate by halophilic PHA-producing bacteria by Dubey and Mishra. The authors proved that *Halomonas daqingensis* and *Halomonas ventosae* were capable of utilizing 3, 4% of algal waste residue and producing 68.96 and 72.41% in the cell dry mass, respectively. Similarly, Borrero-de Acuña et al. used biodiesel-derived crude glycerol in the cultivation of *Pseudomonas putida* KT2440 and its *ΔphaZ* mutant (PHA depolymerase deficient) for the production of mcl-PHAs under fed-batch conditions. Two different feeding approaches were compared to demonstrate that DO-stat fed-batch process is more suitable feeding strategy than the constant-feeding approach resulting in about 50% more PHA at the end of the fermentation (60 h) in the *ΔphaZ* mutant with a specific PHA volumetric productivity of 0.34 g L^−1^ h^−1^. With a reduced fermentation period and unique feeding strategy, *P. putida* strains accumulated higher quantities of biopolymer with a monomeric composition largely consisting of 3-hydroxydecanoate. During the fermentation, citric acid was produced as a major co-product. This provides a scope of further improvement *via* metabolic engineering to decrease the carbon wastage and channelize the carbon flux for PHA biosynthesis.

In the past 2 decades, a lot of efforts have also been made to isolate and explore unique microbes that can produce large quantities of PHAs using cheap raw materials. However, very limited research has been tested on pilot-scale production and translated to produce PHAs on a large-scale. In this regard, photoautotrophic microalgae have recently emerged as one of the potent contenders for cost-effective and sustainable PHA production. They have minimal metabolic requirements, such as inorganic nutrients (CO_2_, N, P, etc.) and light, and they can survive under adverse environmental conditions. Afreen et al. reviewed the potential of microalgae as a microbial factory for PHA production. In addition, PHA production by cyanobacteria (both wild-type and recombinants) such as *Nostoc*, *Arthrospira*, *Synechocystis*, and *Synechococcus* were presented in detail comparing the PHA yield and cultivation conditions. However, there are certain limitations–stringent management of closed photobioreactors, and optimization of monoculture in open pond culture. To overcome these limitations and reduce the feed cost incurred during PHA production, a “Hybrid Biological System” was suggested. It involves the participation of microalgae/cyanobacteria and bacteria to utilize the opportunities present in both photoautotrophic and heterotrophic cultivation with the following cultivation regimes: (A) Mixotrophy; (B) Photoautotrophic-heterotrophic consortium; and (C) Two module system. For the optimization of the process parameters, several relevant metabolic network modeling approaches were also discussed. This review emphasizes the intellectual but efficient use of biological systems and explores various attributes associated with photoautotrophic and heterotrophic organisms.

Similar to the hybrid biological system, open mixed microbial cultures had been favored for PHA production under non-axenic conditions. Such an approach involves enrichment of the PHA producers under specific conditions utilizing organic wastes as substrate. Thus, co-metabolic activities of microbes not only favour microbial growth but also alleviate the substrate cost. Lorini et al. conducted a unique study concerning PHA production at pilot scale exploiting different process configurations and organic waste streams such as the organic fraction of municipal solid waste and sewage sludge, cellulosic primary sludge, and fruit waste. Here, thermal drying and wet acidification of the biomass at the end of the PHA accumulation process were used to preserve the intracellular PHA. The polymer was extracted using aqueous-phase inorganic reagents under optimized conditions. PHA extracted from wet-acidified biomass had higher viscosity average molecular weights compared to thermally stabilized dried biomass. This investigation shows the possibility to exploit organic biowastes as raw materials for PHA production. The stabilization method before extraction helped to preserve the polymer properties. On the other hand, the monomeric composition of the polymer was greatly influenced by the type of feedstock used. This study reveals that waste-derived biopolymers can achieve good characteristics and workability. Such information may play a pivotal role in future development and sustainable PHA production.

Furthermore, Santolin et al. proposed a novel strategy for reducing the dependency of a single feedstock during PHA production process. The authors described effective substrate-flexible two-stage fed-batch cultivations for the production of P(HB-co-HHx) copolymer using fructose during the biomass accumulation stage and rapeseed oil for polymer production. In order to achieve high space time yields a “drain and fill” modus was proposed for semi-continuous biomass production during the initial stage. Taking advantage of the high-cell-density achieved during the first stage, the second stage was run without previous sterilization of the in-series bioreactors. The obtained results suggested that applying the new method presented by the authors could contribute to reduce production costs and, in this way, accelerate the commercialization of a sustainable PHA-bioplastic.

In the field of PHA production, the recovery of the polymer from its intracellular environment is of major importance. This step impact is one of the major contributors to the high selling price of the polymers but also to its sustainability, due to the non-environmentally friendly nature of most of the processes used for its recovery. Delving into this topic, Pagliano and co-workers considered the different natural production of PHA either by pure or mixed cultures and some of the polymer characteristics as well as the utilization of either solvent-based or cellular-lysis-based methods for its recuperation. Parameters as the intracellular PHA content, the PHA recovery and its yield, the PHA purity, molecular weight (M_W_), and polydispersity index (PDI) were used to compare the different methods. Considering the nature of the cultures used there is no rule of thumb to be applied. PHAs produced by MMC are the ones presenting a major variability in the considered parameters mostly due to the different PHA content observed as well as the chemical characteristics of the polymers produced by those cultures. Solvent extraction methods perform better in terms of higher purity and molecular weight of the recovered polymers, mostly for halogenated and carbonate solvents and to a lower extent to ketones and alcohols. Cellular lysis although provides a higher recovery and yield, can cause polymer degradation that will impact both M_W_ and PDI (polymer quality). The purity of the polymer will be affected (70–80%) which would have a major impact on its final utilization. Some techno-economics aspects of the extraction processes were provided that differentiate both methods. In terms of costs, cellular lysis would present lower costs at the expense of a low-quality polymer while solvent extraction, in particular halogenated ones, at a larger scale and considering quality aspects, would become more competitive.

The Guest Editors are thankful to all the submitting authors for considering this Special Issue as a forum for publishing their research work. The assistance of the reviewers, through their high-quality reports, was very helpful for choosing the papers which are suitable for this Special Issue. We hope that this Special Issue will attract the interest of the researchers which are involved in PHAs study and will be an inspiration for them to improve PHA-related processes.
